# Properties of Lithium Trivanadate Film Electrodes Formed on Garnet-Type Oxide Solid Electrolyte by Aerosol Deposition

**DOI:** 10.3390/ma11091570

**Published:** 2018-09-01

**Authors:** Ryoji Inada, Kohei Okuno, Shunsuke Kito, Tomohiro Tojo, Yoji Sakurai

**Affiliations:** Department of Electrical and Electronic Engineering, Toyohashi University of Technology, 1-1 Tempaku-cho, Toyohashi, Aichi 4418580, Japan; okuno@cec.ee.tut.ac.jp (K.O.); kito@cec.ee.tut.ac.jp (S.K.); tojo@ee.tut.ac.jp (T.T.); sakurai@ee.tut.ac.jp (Y.S.)

**Keywords:** aerosol deposition, lithium trivanadate, film electrode, garnet, solid electrolyte

## Abstract

We fabricated lithium trivanadate LiV_3_O_8_ (LVO) film electrodes for the first time on a garnet-type Ta-doped Li_7_La_3_Zr_2_O_12_ (LLZT) solid electrolyte using the aerosol deposition (AD) method. Ball-milled LVO powder with sizes in the range of 0.5–2 µm was used as a raw material for LVO film fabrication via impact consolidation at room temperature. LVO film (thickness = 5 µm) formed by AD has a dense structure composed of deformed and fractured LVO particles and pores were not observed at the LVO/LLZT interface. For electrochemical characterization of LVO film electrodes, lithium (Li) metal foil was attached on the other end face of a LLZT pellet to comprise a LVO/LLZT/Li all-solid-state cell. From impedance measurements, the charge transfer resistance at the LVO/LLZT interface is estimated to be around 10^3^ Ω cm^2^ at room temperature, which is much higher than at the Li/LLZT interface. Reversible charge and discharge reactions in the LVO/LLZT/Li cell were demonstrated and the specific capacities were 100 and 290 mAh g^−1^ at 50 and 100 °C. Good cycling stability of electrode reaction indicates strong adhesion between the LVO film electrode formed via impact consolidation and LLZT.

## 1. Introduction

All-solid-state lithium (Li) ion batteries (LiBs) are expected to be part of the next generation of energy storage devices because of their high energy density, high safety and reliability [1,2,3]. The ceramic materials used as solid electrolytes (SEs) must have, not only high lithium-ion (Li^+^) conductivity above 10^−3^ S cm^−1^ at room temperature, but also deformability and chemical stability against electrode materials, air and moisture. Oxide-based SEs have a relatively low conductivity and poor deformability compared to sulfide-based ones, while they have other advantages, such as chemical stability and ease of handling [4,5,6].

Garnet-type Li-stuffed oxide, Li_7_La_3_Zr_2_O_12_ (LLZ), has been extensively studied because of its good ionic conducting property, excellent thermal performance, and high electrochemical stability [7]. LLZ has two different crystal phases, one is the cubic phase [7,8] and the other is tetragonal one [9,10], but the former has two orders higher conductivity at room temperature than the latter. Partial substitution of the Zr^4+^ site by other higher valence cations, such as Nb^5+^ [11,12] and Ta^5+^ [13,14,15,16,17,18,19] stabilizes the highly-conductive cubic phase. The conductivity at room temperature for both Ta- and Nb-doped LLZ with optimized Li contents (6.4–6.5) in crystal framework attain up to 1 × 10^−3^ S cm^−1^, but the former has much higher chemical stability against Li metal electrode than the latter [20,21].

Another important issue in solid-state batteries with a ceramic ionic conductor, such as SE, is to form good solid–solid interface between the electrode active material and SE, which is indispensable for fast electrochemical reaction in batteries. Although Li-stuffed garnet-type oxide is a good candidate for SE in a solid-state battery, high-temperature sintering at 1000–1200 °C is generally needed for densification [7,11,12,13,14,15,16,17,18,19,20,21] and this temperature is too high to suppress the undesired side reaction between the majority of electrode active materials and SE and the formation of impurity phases [22]. Li^+^ conducting Li_3_BO_3_ with a low melting point (~700 °C) has been applied to form the interface between LiCoO_2_ and garnet-type SE by a co-sintering process [23,24], but the conductivity for Li_3_BO_3_ is low (10^−7^–10^−6^ S cm^−1^ at room temperature) and there are currently limited electrode materials that can be used for solid-state batteries with garnet-type SEs developed by the co-sintering process.

**Figure 1:**
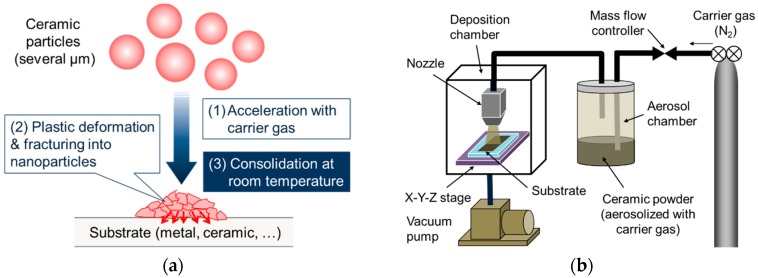
Schematic illustrations for (**a**) film formation mechanism via impact consolidation of ceramic particles and (**b**) aerosol deposition (AD) apparatus.

To address this issue, we have been focusing on the aerosol deposition (AD) method for the fabrication process of the electrode layer in oxide-based solid-state batteries. This method uses impact consolidation at room temperature between ceramic particles and a substrate during aerosolized powder crash onto the substrate (Figure 1a) [25,26,27]. By controlling the size and morphology of the base powder material, the film fabricated by the AD method has a highly-dense structure made of nanocrystalline particles, and the structural and physical properties are similar to the base powder material. This feature is quite attractive in the fabrication of oxide-based solid-state batteries, because various electrode active materials can be selected and formed on an SE with no thermal treatment. Several works for the application of AD to battery materials have been already reported. The electrochemical properties of film-shaped electrodes of LiMn_2_O_4_ [28], Si alloy or composite [29], LiFePO_4_ [30], Li_4_Ti_5_O_12_ [31], LiNi_1/3_Co_1/3_Mn_1/3_O_2_ [32,33], Fe_2_O_3_ [34], TiNb_2_O_7_ [35] and LiNi_0.5_Mn_1.5_O_4_ [36] formed on a metal and a SE substrate are investigated to verify the feasibility of AD. In addition, as-deposited oxide-based SE films with NASICON (Na Superionic Conductor) [37,38], perovskite [39] and garnet-type structure [40,41] show moderate Li^+^ conductivity around 10^−7^–10^−5^ S cm^−1^ at room temperature.

In this work, we fabricated a lithium trivanadate LiV_3_O_8_ (LVO) film electrode by AD onto a garnet-type Ta-doped LLZ (Li_6.55_La_3_Zr_1.55_Ta_0.45_O_12_, LLZT) SE for the first time. LVO has been studied for a long time as a cathode active material for rechargeable Li-based batteries [42,43,44,45,46,47,48,49], because of the large Li^+^ storage capacity of 300 mAh g^−1^ at an averaged potential around 2.5 V vs. Li/Li^+^. It is noted that the reaction of LVO starts from discharging (i.e., Li^+^ insertion) process, which is different from other conventional cathode materials for LiBs such as LiCoO_2_, LiMn_2_O_4_ and LiFePO_4_ which contain Li^+^ for the charge and discharge reaction. Therefore, an anode material in a rechargeable battery with LVO cathode must contain Li^+^ used for the charge and discharge reaction, which means a graphite anode is difficult to use in combination with LVO cathode. In solid-state batteries with garnet-type SE, Li metal electrodes may potentially be used as anodes; thus, LVO would become an attractive candidate for a high capacity cathode. Ball-milled LVO powder with a size of 0.5–2 µm was used as the raw material for film fabrication on glass, SUS316L and LLZT pellets as substrates via impact consolidation at room temperature. The crystal phase and microstructure of LVO film and LVO/LLZT interface were evaluated. For the electrochemical characterization of the LVO film electrode, a Li metal foil was attached on the opposite end face of the LLZT pellet as an anode to form an LVO/LLZT/Li all-solid-state cell. The electrochemical properties of the solid-state cell were investigated by a galvanostatic charge and discharge testing.

## 2. Materials and Methods

### 2.1. Synthesis and Characterization of LVO Powder Used for Film Fabrication

LVO powder was prepared by a conventional solid-state reaction process. The following were obtained from the Kojundo Chemical Laboratory (Saitama, Japan): Stoichiometric amounts of LiOH•H_2_O (99%) and V_2_O_5_ (99.9%), which were then ground and mixed in an agate mortar for 0.5 h with acetone, and then calcined at 570 °C for 10 h in air using an Al_2_O_3_ crucible.

It is known that the controlling of both the size and morphology of raw powder are important for film fabrication via impact consolidation [25,26,27,33,38]. In order to prepare LVO powders suitable for the film fabrication, as-synthesized LVO powder was pulverized using planetary ball-milling (Nagao System, Planet M2-3F, Kawasaki, Japan) with ethanol and zirconia balls (2 mm in diameter) for 1 h in a zirconia pot. The rotation speed of the planetary ball-milling was set to 250 rpm.

The particle size distributions for LVO powders were evaluated using a Laser Diffraction Particle Size Analyzer (SHIMADZU, SALD-2000, Kyoto, Japan). The crystal phase of LVO powder was evaluated by an X-ray diffractometer (XRD; RIGAKU, MultiFlex, Tokyo, Japan) using CuKα radiation (λ = 0.15418 nm), with a measurement range 2*θ* of 5–90° and a step interval of 0.004°. A field emission scanning electron microscope (FE-SEM; Hitachi High-Technologies, SU8000 Type II, Tokyo, Japan) was used to observe the morphology and size of the LVO powders.

### 2.2. Synthesis and Characterization of Garnet-Type LLZT Pellet

LLZT pellets were prepared using a conventional solid-state reaction process reported in our previous work [19,35]. All starting materials were obtained from the Kojundo Chemical Laboratory (Saitama, Japan): stoichiometric amounts of LiOH·H_2_O (99%, 10% excess was added to account for the loss of Li at high temperatures), La(OH)_3_ (99.99%), ZrO_2_ (98%) and Ta_2_O_5_ (99.9%), which were then pulverized and mixed by planetary ball-milling with zirconia balls (5 mm in diameter) and ethanol for 3 h in a zirconia pot, and then calcined at 900 °C for 6 h in air using a Pt-5% Au alloy crucible. The calcined powders were ball-milled again for 1 h and then pelletized under the pressure of 300 MPa using cold isostatic pressing. Finally, they were sintered at 1150 °C for 15 h in air using a Pt-5% Au alloy crucible. To minimize Li loss and the formation of impurities during the sintering, the pellets were covered with the same mother powder.

From XRD measurement and FE-SEM observation, we confirmed that LLZT has a cubic garnet structure without any impurity phases and a dense structure composed of LLZT grains with an average size of 5 µm (Figure S1). The conductivity at 27 °C and activation energy for LLZT were confirmed to be 9 × 10^−4^ S cm^−1^ and 0.40 eV [19].

### 2.3. Fabrication and Characterization of LVO Films by AD on Glass, SUS316L and LLZT

As shown in Figure 1b, AD apparatus consists of a carrier gas supplying system, an aerosol chamber, a deposition chamber equipped with a motored *X-Y-Z* stage and a nozzle with an orifice with a thin rectangular shape (10 mm × 0.5 mm). Nitrogen (N_2_) gas was used as a carrier gas. N_2_ gas flows out from a gas supply system to an aerosol chamber and powder in the aerosol chamber is dispersed into the carrier gas. The deposition chamber was evacuated to a low vacuum state around 20 Pa. Finally, well-dispersed aerosol flows into the deposition chamber through a nozzle and is sprayed onto a substrate, by the difference of pressures in an aerosol chamber and a deposition chamber. The deposition area was masked into a circular shape with a diameter of 8 mm. Deposition was carried out for 5–10 min and during the deposition process, the stage was moved uni-axially with a back-and-forth motion length of 50 mm and a speed of 10 mm s^−1^. According to our previous works [33,35,38], the distance between a nozzle tip and a substrate and a flow rate of N_2_ gas was set to 10 mm and 20 L min^−1^.

In order to investigate LVO powder suitability for film fabrication via impact consolidation, both as-synthesized and ball-milled powders were used as raw materials. Glass, SUS316L and LLZT pellets were used as substrates. The crystal phase of LVO films formed on a glass plate and a LLZT pellet were evaluated by XRD using CuKα radiation, with a measurement range 2*θ* of 5–90° and a step interval of 0.004°. The microstructure of LVO films that formed on a glass plate and a LLZT pellet was observed by FE-SEM. Energy dispersive X-ray (EDX) analysis was also performed using FE-SEM, to investigate the fractured surface microstructure of the LVO film formed on LLZT and the corresponding distribution of V, La and Zr elements.

### 2.4. Electrochemical Characterization for LVO Film Formed on LLZT Solid Electrolyte

For electrochemical characterization of the LVO film electrode formed on LLZT, we constructed a LVO/LLZT/Li all-solid-state cell with a cell fixture in an Ar-filled grove box (Figure S2), by attaching a Li metal foil on the polished end surface of an LLZT pellet with 1.90 mm thickness. Before the cell construction, an Au film as a current collector was deposited onto the LVO film by sputtering. A heat treatment at 175 °C for 5 h was applied to the cell after the cell construction, to reduce the interfacial charge–transfer resistance *R*_Li-LLZT_ between Li and LLZT [19].

The electrochemical impedance for the LVO/LLZT/Li cell was measured at 27 °C with a chemical impedance meter (HIOKI, 3532-80, Ueda, Japan) at frequencies from 5 to 10^6^ Hz and an applied voltage amplitude of 0.05 V. Charge and discharge properties of the LVO film electrode in a solid-state cell were investigated by a galvanostatic test at 50 °C and 100 °C using a Battery Test System (TOYO SYSTEM, TOSCAT-3100, Iwaki, Japan). Current densities for galvanostatic testing were changed in the range of 0.004–0.240 mA cm^−2^ (corresponding to 5–300 mA g^−1^ for the LVO film electrode).

## 3. Results and Discussion

### 3.1. Characterization of LVO Powders and Films on Glass and SUS316L Plates

**Figure 2:**
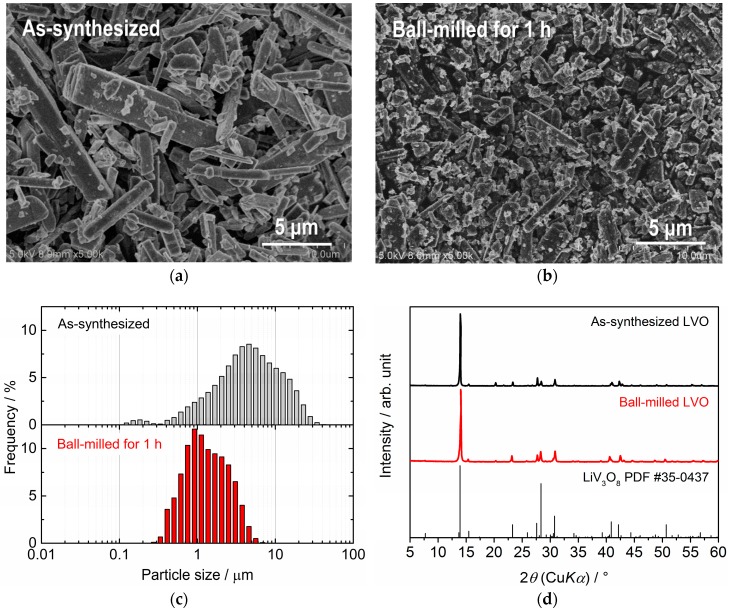
Scanning electron microscope (SEM) images of (**a**) as-synthesized and (**b**) ball-milled LiV_3_O_8_ (LVO) powders. Comparisons of particle size distributions and X-ray diffraction (XRD) patterns for both powders are also shown in (**c**) and (**d**).

Figure 2a,b are scanning electron microscope (SEM) images for as-synthesized and ball-milled LVO powders. Most of the LVO particles in as-synthesized powder have rod-like morphology with non-uniform length from 5 to 15 µm and thickness from 0.5 to 1.5 µm. After the ball-milling, most of the large rod-shaped particles are well pulverized to 1–2 µm, but some of the small rod-shaped particles with a length around 5 µm still remained without pulverization. These features are consistent with the particle size distribution measurements (Figure 2c). XRD patterns for both LVO powders are compared in Figure 2d. Noticeable changes in the patterns and the peaks from other phases are not confirmed after the ball-milling process, indicating that LVO particles were pulverized without any structural changes.

**Figure 3:**
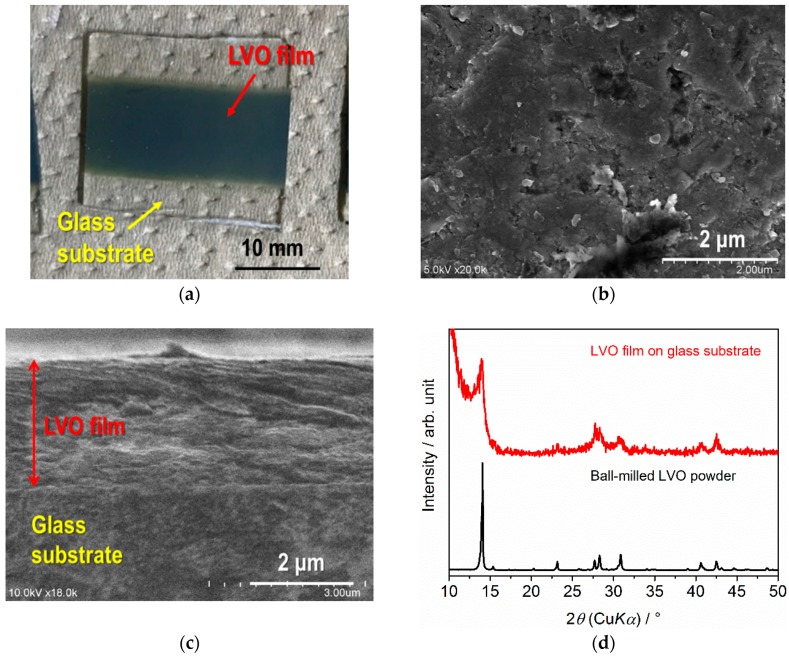
(**a**) Photo of the LVO film formed on a glass substrate by AD. (**b**) and (**c**) are SEM images for the surface and fractured cross-section of the LVO film on a glass substrate. XRD patterns for the LVO film formed on a glass substrate by AD and LVO powder are compared in (**d**).

Although both as-synthesized and ball-milled LVO powders were used as raw materials for film fabrication by AD on glass and SUS316L substrates, only the latter (Figure 2b) was confirmed to be suitable to form a film on each substrate. We are considering that as-synthesized powder is too large to form the film via impact consolidation. Figure 3a,b show the photo of the LVO film formed on a glass substrate and a SEM image of the surface of the LVO film. It is confirmed that LVO powders are strongly deformed or fractured to form the film by impact consolidation. From the mass and thickness of the film confirmed by SEM observation (Figure 3c), the relative density of LVO film formed by AD is confirmed to be approximately 85%.

XRD patterns for the LVO film deposited on a glass substrate are shown in Figure 3d and compared with the data for LVO powders used for the film fabrication. The diffraction peaks from the LVO film are clearly confirmed, indicating that crystalline LVO film was successfully fabricated with no thermal treatments. The peaks from other secondary phases were not observed, but the peaks for LVO become broader than those for the powder used for AD. A similar phenomenon has been confirmed in other ceramic films formed by AD [25,26,32,34,35,36,37,38,39,40,41]. This could be attributed to the degradation of the crystallinity and/or the atomization of LVO particles during the impact consolidation.

We also checked the electrochemical performance for the LVO film electrode in a liquid organic electrolyte by galvanostatic charge and discharge testing for a two electrode set-up. LVO film on a SUS316L substrate is used as a working electrode, while single Li foil is used as counter and reference electrodes. As shown in Figure S3, the LVO film electrode shows a reversible charge and discharge reaction with a specific capacity around 300 mAh g^−1^ at 25 °C and 30 mA g^−1^ (=0.1 C), which is comparable with a LVO composite electrode with a conducting additive and a binder [42,43,44,45,46,47,48,49]. In addition, we observed the stage-like behavior of the charge/discharge curves in LVO film electrode, referring to the different oxidation states of vanadium.

### 3.2. Characterization for LVO Film Electrode Formed on LLZT Solid Electrolyte

**Figure 4:**
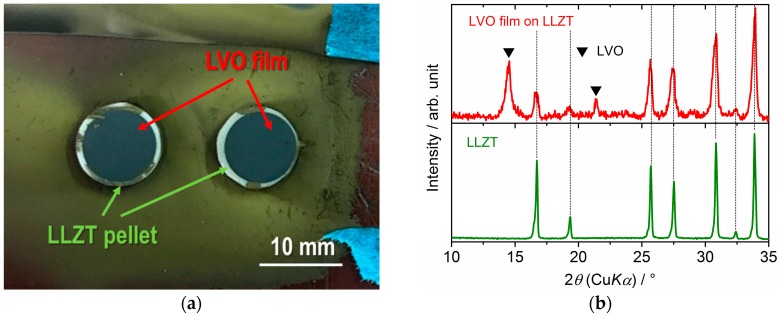
(**a**) Photo and (**b**) XRD pattern for LVO film formed on a Ta-doped Li_7_La_3_Zr_2_O_12_ (LLZT) pellet by AD.

**Figure 5:**
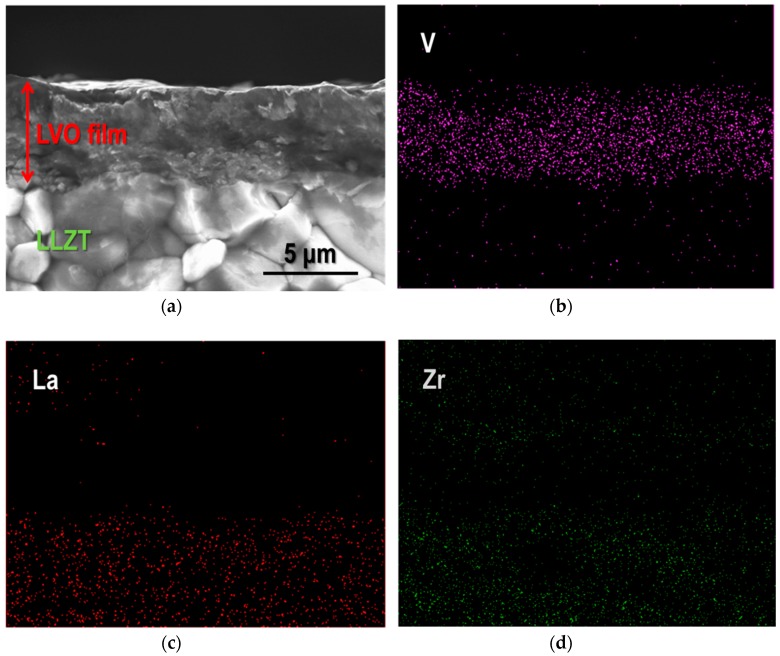
(**a**) SEM image for a fractured cross-sectional surface of the LVO film formed on LLZT by AD, and the corresponding elementary mapping are also shown for (**b**) V, (**c**) La and (**d**) Zr elements.

Based on the previously mentioned results, we tried to fabricate a LVO film on a LLZT pellet by AD using a ball-milled LVO powder (Figure 2b) and the film was formed successfully on LLZT, as well as glass and SUS316L substrates. Figure 4a,b show the photo and the XRD pattern of LVO film on LLZT pellet by AD. The diffraction peaks from both the LVO film and the LLZT pellet were clearly observed. A cross-sectional SEM image and corresponding elementary mapping for V, La and Zr are shown in Figure 5. Dense LVO film is solidified on LLZT and the interface between LVO and LLZT is smooth (Figure 5a). Pores are hardly confirmed in both LVO film and LVO/LLZT interface. Although the elementary distribution of Zr (Figure 5d) is slightly ambiguous, the distributions of V (Figure 5b) and La (Figure 5c) reflect well the laminated structure of LVO film and LLZT. The reason for the ambiguous distribution of Zr has not been clarified as of yet but it is possibly due to the background noise.

**Figure 6:**
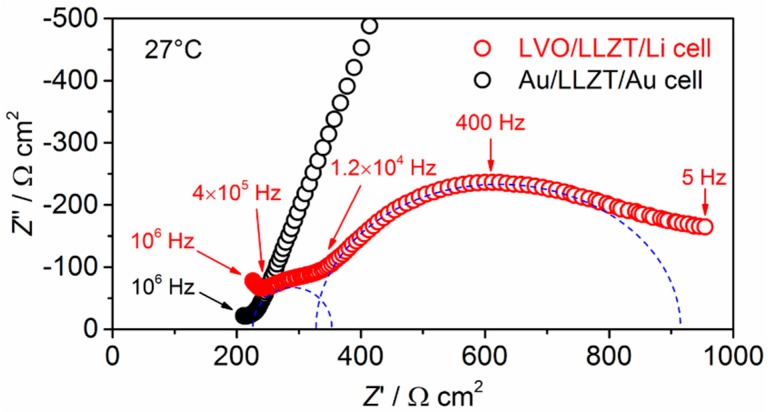
Nyquist plots for electrochemical impedance at 27 °C in LVO/LLZT/Li and Au/LLZT/Au cells.

Figure 6 shows the Nyquist plot for an electrochemical impedance at 27 °C for a LVO/LLZT/Li all-solid-state cell (after heat treatment at 175 °C for 5 h) and frequency from 5 to 10^6^ Hz. For comparison, the data for a LLZT pellet with Li^+^ blocking Au electrodes (i.e., Au/LLZT/Au symmetric cell) is also plotted. Compared to the data for Au/LLZT/Au symmetric cell, the area specific resistance of LLZT is confirmed to be 210–220 Ω cm^2^. A large semi-circle from 5 Hz to 1.2 × 10^4^ Hz and a smaller one from 10^4^ Hz to 4 × 10^5^ Hz were observed in LVO/LLZT/Li cell. In our previous work [19], charge transfer resistance at Li/LLZT interface *R*_Li-LLZT_ at 27 °C is reduced below 100 Ω cm^2^ by a heated Li/LLZT/Li symmetric cell at 175 °C for 3 to 5 h, and the characteristic frequency for charge transfer at a Li/LLZT interface is around 10^4^–10^5^ Hz. By addressing them, the smaller semi-circle at a higher frequency range corresponds to *R*_Li-LLZT_ while the larger semi-circle at lower frequency range indicates the contribution from the LVO/LLZT interface. Charge transfer resistance at LVO/LLZT interface is estimated to be approximately 600 Ω cm^2^ at 27 °C.

**Figure 7:**
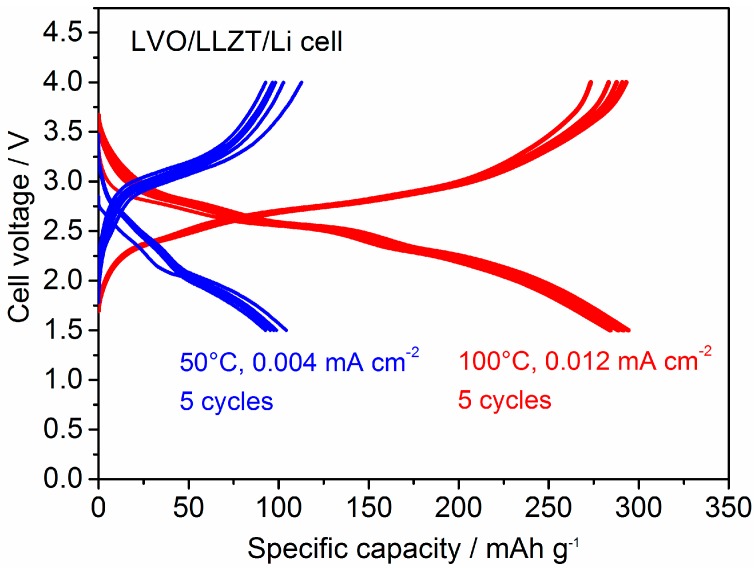
Comparison of galvanostatic charge and discharge curves for the LVO/LLZT/Li solid-state cell measured at 50 °C and 0.004 mA cm^−2^ (=0.0167 C) and 100 °C and 0.012 mA cm^−2^ (=0.05 C). The measurements at each temperature are repeated for five cycles.

The galvanostatic charge (Li^+^ extraction form LVO) and discharge (Li^+^ insertion into LVO) curves for five cycles in an LVO/LLZT/Li cell measured at 50 °C and 100 °C are shown in Figure 7. The current density is fixed to 0.004 mA cm^−2^ (corresponding to 5 mA g^−1^ (=0.0167 C)) at 50 °C and 0.012 mA cm^−2^ (corresponding to 15 mA g^−1^ (=0.05 C)) at 100 °C. Reversible charge and discharge reactions in the LVO film electrode in the solid-state cell were confirmed at each temperature. The specific capacity of 100 mAh g^−1^ was obtained at 50 °C, but the polarization seems to be very large. This could be attributed to both large *R*_LVO-LLZT_ and slow Li^+^ diffusion in the film composed of deformed and fractured LVO nanoparticles. Both the electronic and ionic conductivity of LVO are reported to be around 10^−7^ S cm^−1^ at room temperature [49]. Moreover, a LVO film formed by AD has many grain boundaries among the fractured LVO nanoparticles, which may cause large grain boundary resistance [34,35,36,37,38,39,40,41] and prevent the transport of electrons and Li^+^ in the film. In order to distinguish between the percolation limitation of the cathode film and the cathode/electrolyte interface, the electrical conducting properties of a LVO film formed by AD should be investigated further in the future. With increasing the temperature to 100 °C, the polarization is greatly reduced and the capacity increases significantly to 290 mAh g^−1^ at an averaged cell voltage around 2.5 V. Furthermore, the stage-like behavior of the charge/discharge curves due to the different oxidation states of vanadium is also visible in LVO film electrode formed on LLZT, which is also observed in a typical behavior for LVO composite electrode in an organic liquid electrolyte [42,43,44,45,46,47,48,49]. Although the high temperature is needed to obtain better electrochemical performance at present, to the best of our knowledge, this is the first demonstration of applying a LVO electrode in an oxide-based all-solid-state cell.

**Figure 8:**
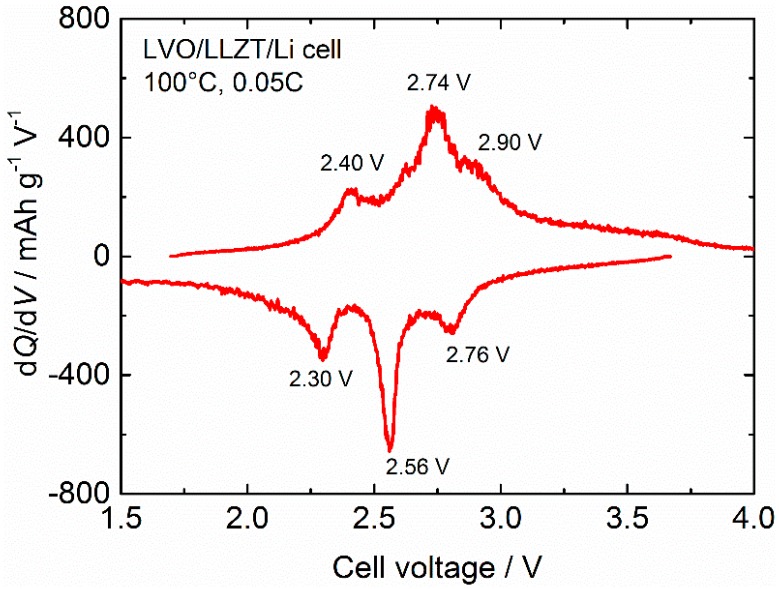
d*Q*/d*V* (*Q*: specific capacity, *V*: cell voltage) curve for LVO/LLZT/Li solid-state cell at 100 °C and 0.05 C.

For further examination of the electrochemical reaction in the LVO film electrode on LLZT, d*Q*/d*V* (*Q* and *V* are the specific capacity and cell voltage) curve for LVO/LLZT/Li cell at 100 °C and 0.012 mA cm^−^^2^ (=0.05 C) is shown in Figure 8. Three main cathodic peaks (at 2.40, 2.74 and 2.90 V) and three main anodic ones (at 2.30, 2.56 and 2.76 V) are clearly confirmed in the d*Q*/d*V* curve, which are attributed to several phase transformations between the couples of Li_1 + x_V_3_O_8_ (x = 0.1–3) [42,43,44,45,46,47,48,49].

**Figure 9:**
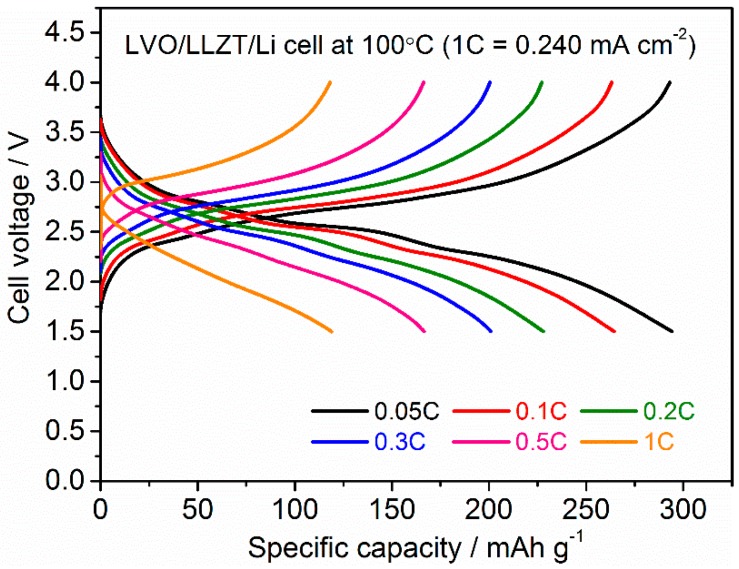
Charge and discharge curves for LVO/LLZT/Li solid-state cell and 100 °C and different current densities from 0.015 to 0.240 mA cm^−2^. Note that 1 C rate (=30 mA g^−1^) corresponds to 0.240 mA cm^−2^.

**Figure 10:**
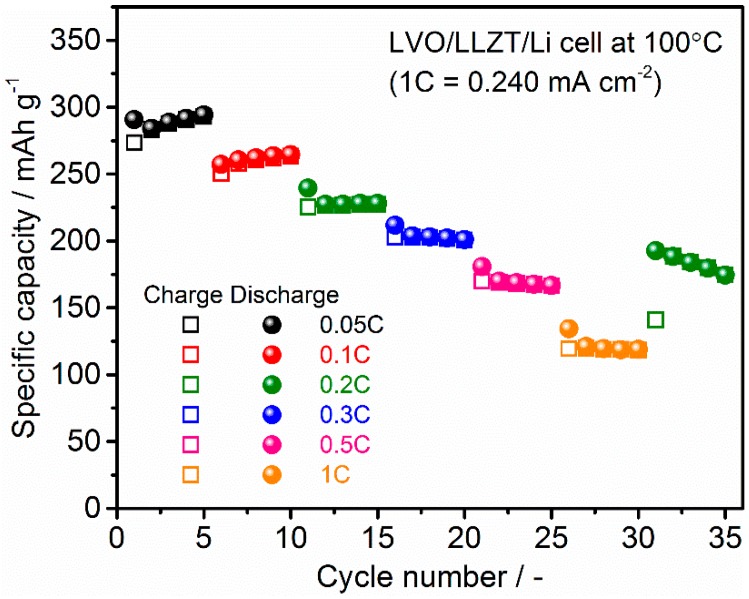
Cycling performance of charge and discharge capacities for LVO/LLZT/Li solid-state cell at 100 °C and different current densities from 0.015 to 0.240 mA cm^−2^. Note that 1 C rate (=30 mA g^−1^) corresponds to 0.240 mA cm^−2^.

Figure 9 shows the charge and discharge performance in a LVO/LLZT/Li cell at 100 °C and different current densities of 0.012–0.240 mA cm^−2^ (corresponding to 15–300 mA g^−1^ for LVO film). As can be seen, the polarization in both charge and discharge reactions becomes large and reversible capacities are reduced gradually with increasing current densities: 270 mAh g^−1^ at 0.024 mA cm^−2^ (=0.1 C), 230 mAh g^−1^ at 0.048 mA cm^−2^ (=0.2 C), 205 mAh g^−1^ at 0.072 mA cm^−2^ (=0.3 C), 170 mAh g^−1^ at 0.120 mA cm^−2^ (=0.5 C) and 120 mAh g^−1^ at 0.240 mA cm^−2^ (=1 C). As shown in Figure 10 and Figure S4, charge and discharge reactions are stably cycled at each current density. This could be attributed to strong adhesion between an LVO film electrode and LLZT and LVO particles in the film.

As a future prospect, a composite structure with electrode active material and SE is needed to increase the solid–solid interface among them for the high utilization of active material in a thicker composite electrode. The use of composite powders with an electrode active material and a Li^+^ conducting NASICON-type SE as raw materials for electrode fabrication by AD is proposed, to make a solid–solid interface between electrode active material and SE in a thicker electrode layer [33,36]. However, the room temperature conductivity in as-deposited NASICON-type SE films by AD is reported to be only around 10^−6^ S cm^−1^ [37,38]. Since the ceramic particles are plastically deformed and consolidated in the AD process, the use of oxide-based SE materials with both good Li^+^ conduction property and deformability [50,51] is the key to form a better solid–solid interface in the composite electrode by AD. We are now trying to form a thicker composite electrode with LVO as an active material on LLZT by AD, and the progress will be reported in a forthcoming paper.

## 4. Conclusions

We fabricated a lithium trivanadate LVO film electrode using the AD method for the first time on a garnet-type LLZT solid electrolyte. Ball-milled LVO powders with a particle size of 0.5–2 µm are suitable for film fabrication by AD. LVO film (thickness = 5 µm) formed by AD has a dense structure composed of deformed or fractured LVO particles and pores were not observed at LVO/LLZT interface. Reversible charge and discharge reactions in the LVO/LLZT/Li solid-state cell were demonstrated and at a low current rate, the specific capacities of 100 and 290 mAh g^−1^ at 50 and 100 °C were obtained. The cycling stability in LVO/LLZT/Li cell indicates strong adhesion between the LVO film electrode and LLZT and LVO particles in the film.

## Supplementary Materials

The following are available online at http://www.mdpi.com/1996-1944/11/9/1570/s1, Figure S1: XRD patterns and SEM image for sintered Li_6.55_La_3_Zr_1.55_Ta_0.45_O_12_ (LLZT) used in this work, Figure S2: Illustration (left) and photo of cell fixture for composing LVO/LLZT/Li all-solid-state cell, Figure S3: Galvanostatic charge and discharge curves for LVO film electrode (thickness = 2.5 µm) formed on a SUS316L plate in organic liquid electrolyte, Figure S4: Galvanostatic charge and discharge curves for a LVO/LLZT/Li solid-state cell at 100 °C and different current densities.
