# Supplementary material for: Properties of Lithium Trivanadate Film Electrodes Formed on Garnet-Type Oxide Solid Electrolyte by Aerosol Deposition
Source: Materials (Basel). 2018 Sep 1;11(9):1570. doi: 10.3390/ma11091570 (PMC6164746; doi:10.3390/ma11091570)
Supplement: Supplementary file 1 [file materials-11-01570-s001.pdf]

Supplementary Information for
Properties of Lithium Trivanadate Film Electrode Formed on Garnet- Type Oxide Solid Electrolyte by Aerosol Deposition

Ryoji Inada *, Kohei Okuno, Shunsuke Kito, Tomohiro Tojo and Yoji Sakurai

Department of Electrical and Electronic Information Engineering, Toyohashi University of Technology, 1-1 Hibarigaoka, Tempaku-cho, Toyohashi, Aichi 441-8580, Japan

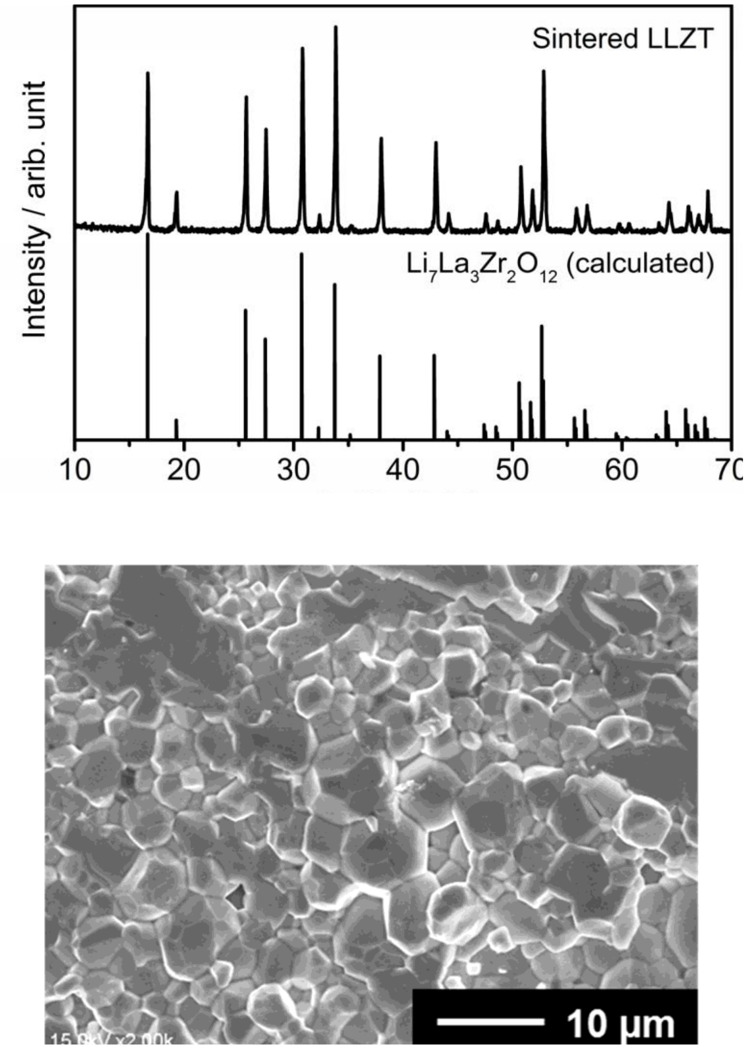

Figure S1. X-ray diffractometer (XRD) patterns (**top**) and scanning electron microscope (SEM) image (**bottom**) for sintered $Li_{6.55}La_3Zr_{1.55}Ta_{0.45}O_{12}$ (LLZT) used in this work. LLZT has a cubic garnet structure without any impurity phases and a dense structure composed of LLZT grains with an average size of 5 μm.

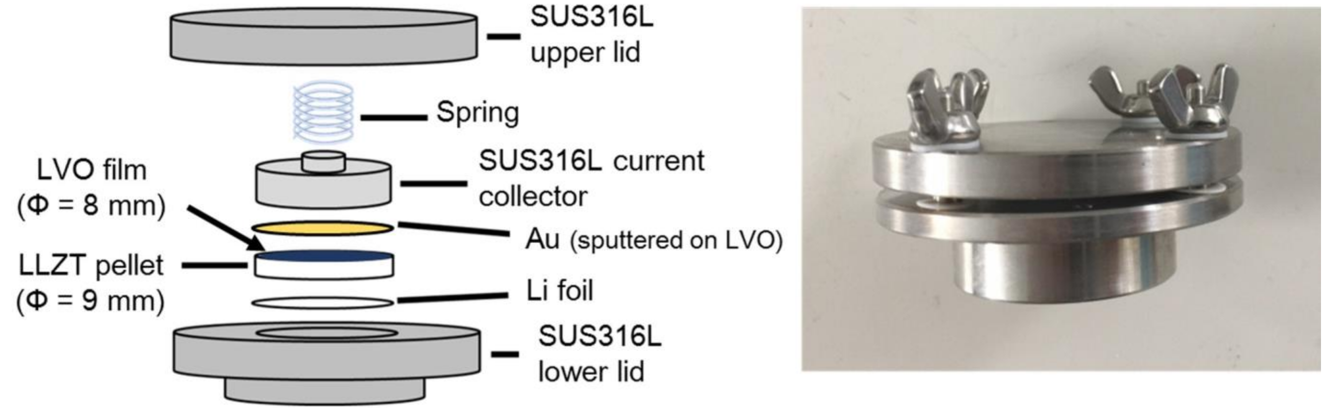

Figure S2. Illustration (**left**) and photo (**right**) of cell fixture for composing a LiV3O8 (LVO)/LLZT/Li all-solid-state cell sample.

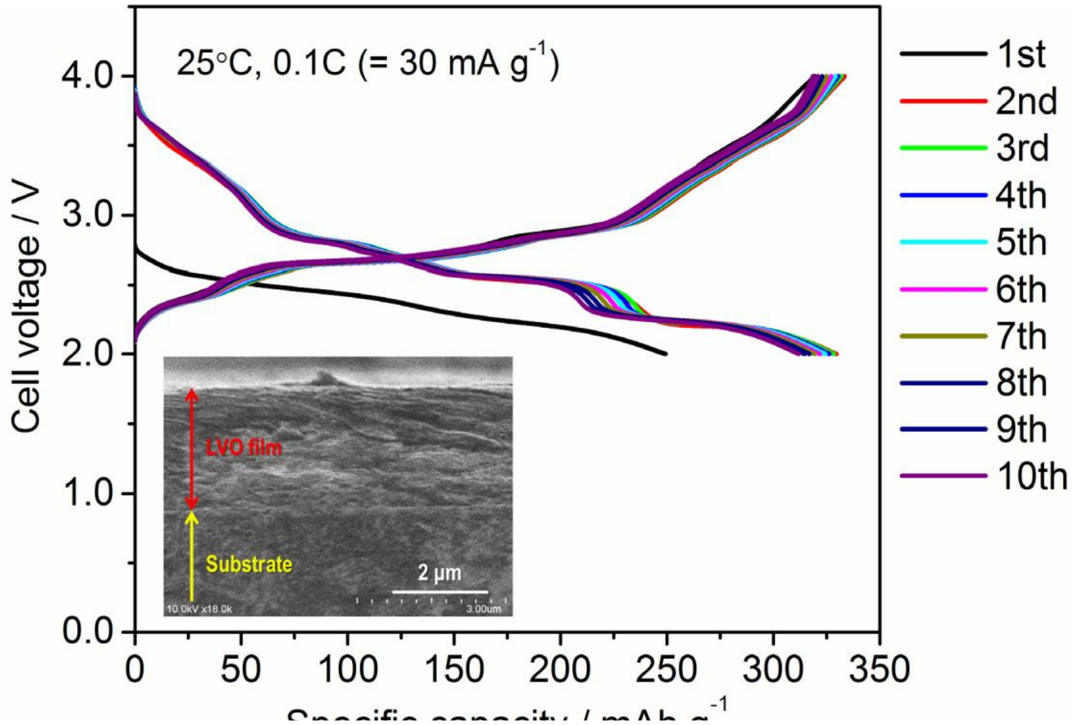

Figure S3. Galvanostatic charge and discharge curves for LVO film electrode (thickness = 2.5 m) formed on a SUS316L plate in an organic liquid electrolyte. LVO film is used as working electrode, where as a single Li foil serves as both counter and reference electrodes. The electrolyte solution was 1 mol $LiPF_6$ in a mixture or ethylene carbonate (EC) and dimethyl carbonate (DMC) with a volume ratio of 1:1. Together with Celgard 3501 as a separator, these components were assembled in a CR2032 coin type cell in a dry Ar filled grove box.

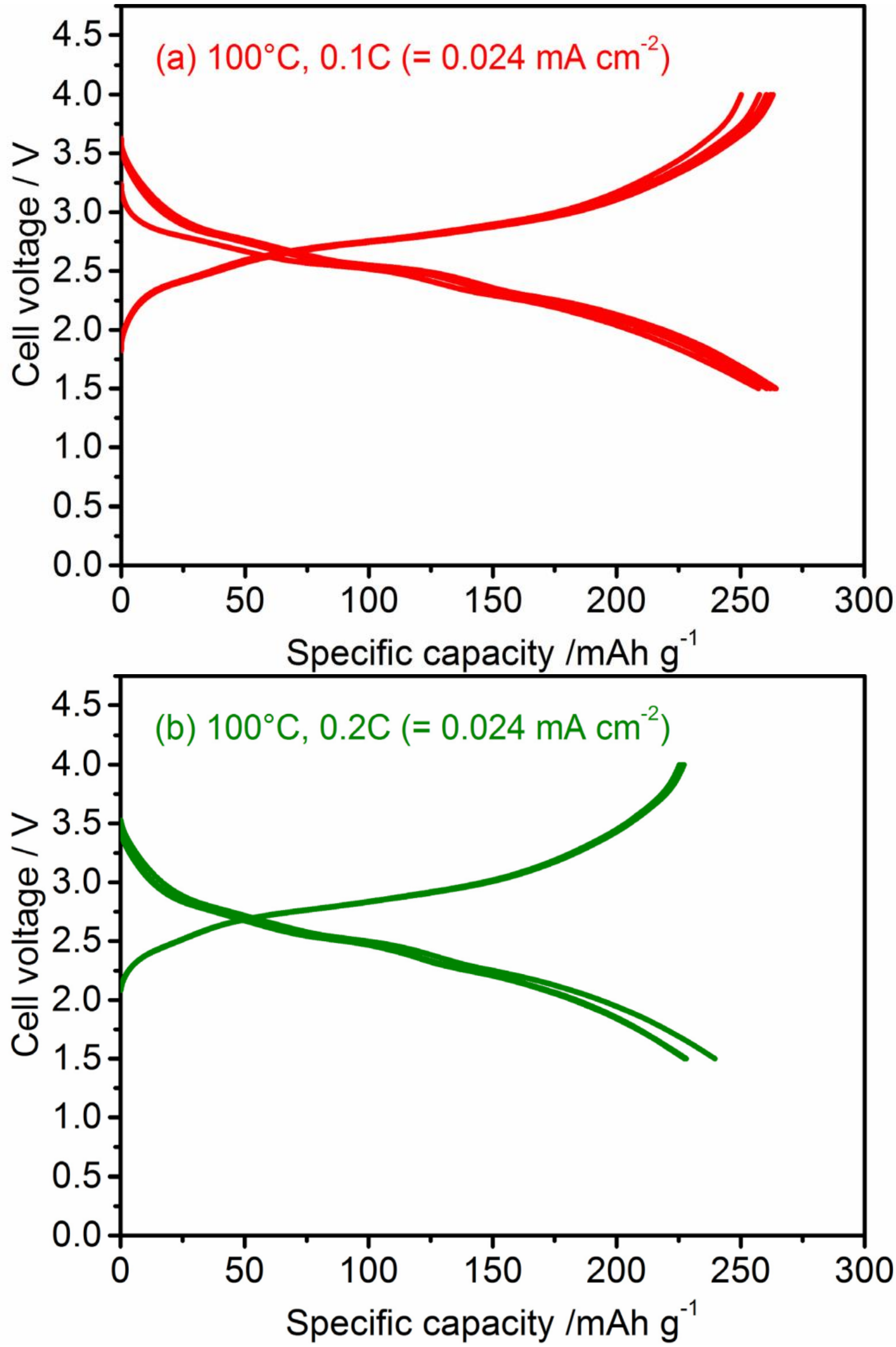

Figure S4. Galvanostatic charge and discharge curves for a LVO/LLZT/Li solid-state cell at 100 C and different current densities: (**a**) 0.030 mA cm^{-2}, (**b**) 0.060 mA cm^{-2}, (**c**) 0.090 mA cm^{-2}, (**d**) 0.120 mA cm^{-2} and (**e**) 0.240 mA cm^{-2}. The measurement at one specific current density is repeated for five cycles. Note that current density of 0.030 mA cm^{-2} corresponds to 30 mA g^{-1} for the LVO electrode.

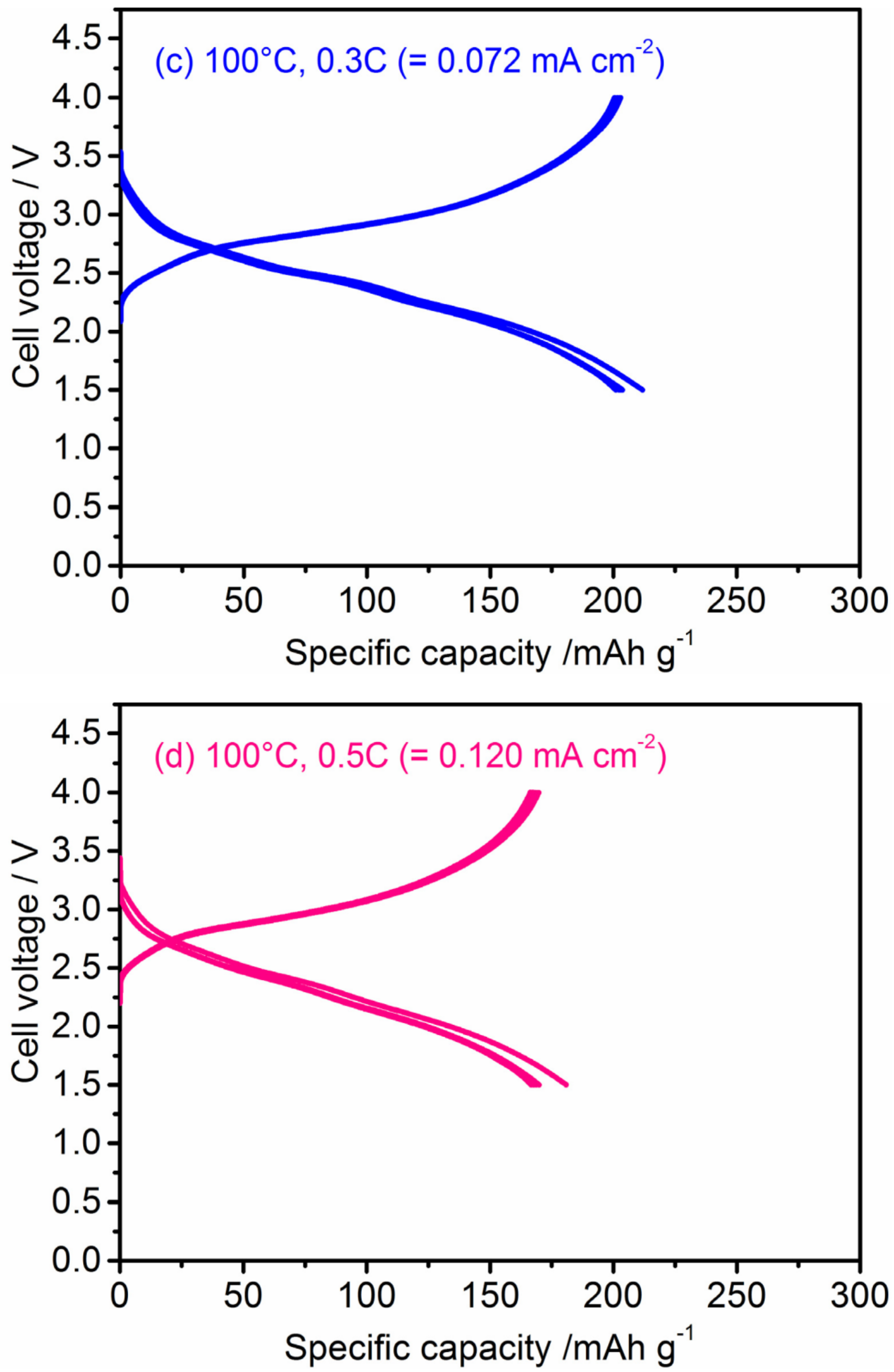

Figure S4. (Continued).

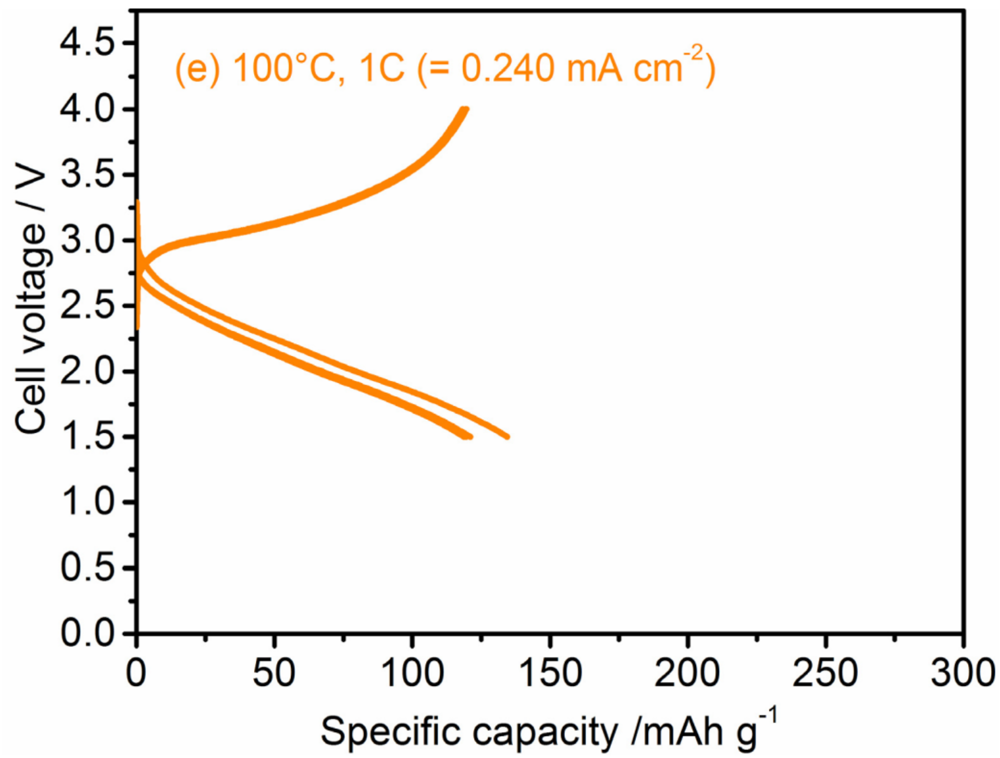

Figure S4. (Continued).
